# Xipho-Omphalopagus Conjoined Twins in a Spontaneous Triplet Pregnancy: Autopsy Findings

**Published:** 2013-11-18

**Authors:** Afifa Sellami, Nozha Chakroun, Rim Frikha, Nouha Abdelmoula Bouayed, Habib Amouri, Tarek Rebai

**Affiliations:** Histology-Embryology Laboratory and Research Unit, Medical School Sfax Tunisia; Histology-Embryology Laboratory and Research Unit, Medical School Sfax Tunisia; Histology-Embryology Laboratory and Research Unit, Medical School Sfax Tunisia; Histology-Embryology Laboratory and Research Unit, Medical School Sfax Tunisia; Gynaecology and obstetrics department, Hedi Chaker academic hospital Sfax Tunisia; Histology-Embryology Laboratory and Research Unit, Medical School Sfax Tunisia

**Dear Sir,**

Conjoined twins (CTs) occur in 1 per 50,000 to 100,000 live births with a female predominance (F:M, 3:1).[1-3] It is exceptionally rare in triplet pregnancies. The aim of this report is to describe autopsy findings of conjoined twins in a spontaneous triplet pregnancy diagnosed by ultrasound prenatally.

A 20-year-old woman, gravid-1 para-0, was referred to the Gynaecology and Obstetrics Department at 21-weeks gestation with a diagnosis of CT in a spontaneous triplet pregnancy. There was no history of twinning in the family and medication use during early pregnancy. At referral, transvaginal ultrasound revealed a monochorionic-diamniotic triplet pregnancy with a set of CTs. One amniotic sac contained a single female foetus; the second amniotic sac had a set of CT fused through the thorax and abdomen. They had two heads, separate hearts and stomachs, two pelvises, four arms and four legs. An extensive counselling of the parents was done regarding the management options. The parents opted termination of pregnancy. Autopsy revealed a normal female foetus and a set of CTs (Fig. 1).The conjoined foetuses were xipho-omphalopagus, joined in midline, from the lower part of the sternum to the umbilicus. They shared a single umbilical cord with two umbilical arteries and two umbilical veins. They had separate chests, separate hearts and lungs with a continuous diaphragm. They shared a common centrally placed fused liver. The intestines were also fused from the mid portion of duodenums to distal ileum. Normal kidneys, urinary bladders, and female genital tracts were noted in each of the twins.

**Figure:**
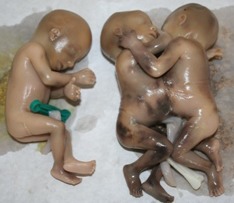
Figure 1: Three female foetuses with a set of conjoined twins.

Embryology of CTs is explained by fission theory which proposes incomplete separation of the inner cell mass of a monozygotic twins; and fusion theory based on the secondary union of two originally separate monozygotic embryonic discs.[1,4] According to another hypothesis CTs result from the development of two co-dominant notochords during gastrulation.[4] Moreover, factors that induce calcium depression and delayed implantation encourage uniovular duplication in general and conjoined twinning in particular.[5] This hypothesis could explain the occurrence of CTs in our case as the mother was from a low socio-economical background with decreased calcium intake in diet. This may be a reason for triplet pregnancy as well as conjoined twinning. Other possible teratogenic factors are oral contraceptives, clomiphene, and oral griseofulvin.[6]

CTs are generally classified into three major groups: twins with a ventral union {cephalopagus (head), thoracopagus (chest), omphalopagus (umbilicus) and ischiopagus (hip)}, twins with a dorsal union { pygopagus (sacrum), rachipagus (spine) and craniopagus (cranium)}, and twins with a lateral union { parapagus (side) twins}.[4] The most frequent varieties of CTs are thoracopagus (40%), omphalopagus (33%) and pygopagus (18%).[3] The prognosis of CTs depends on fusion site, complexity of shared organs, and accompanying anomalies. Approximately 40% were stillborn. Among 60% live born, 35 % have abnormalities incompatible with life and die within 24 hours.[2] Eventually, the overall survival rate for conjoined twins is approximately 18%.[2] CTs pregnancies pose a significant medical challenge with reference to management and counseling.

## Footnotes

**Source of Support:** Nil

**Conflict of Interest:** None declared

